# Development and Validation of a Deep Learning Model Using Convolutional Neural Networks to Identify Scaphoid Fractures in Radiographs

**DOI:** 10.1001/jamanetworkopen.2021.6096

**Published:** 2021-05-06

**Authors:** Alfred P. Yoon, Yi-Lun Lee, Robert L. Kane, Chang-Fu Kuo, Chihung Lin, Kevin C. Chung

**Affiliations:** 1 Section of Plastic Surgery, Department of Surgery, University of Michigan Medical School, Ann Arbor; 2 Center for Artificial Intelligence in Medicine, Chang Gung Memorial Hospital, Taipei, Taiwan; 3 Chang Gung Memorial Hospital, Taipei, Taiwan

## Abstract

**Importance:**

Scaphoid fractures are the most common carpal fracture, but as many as 20% are not visible (ie, occult) in the initial injury radiograph; untreated scaphoid fractures can lead to degenerative wrist arthritis and debilitating pain, detrimentally affecting productivity and quality of life. Occult scaphoid fractures are among the primary causes of scaphoid nonunions, secondary to delayed diagnosis.

**Objective:**

To develop and validate a deep convolutional neural network (DCNN) that can reliably detect both apparent and occult scaphoid fractures from radiographic images.

**Design, Setting, and Participants:**

This diagnostic study used a radiographic data set compiled for all patients presenting to Chang Gung Memorial Hospital (Taipei, Taiwan) and Michigan Medicine (Ann Arbor) with possible scaphoid fractures between January 2001 and December 2019. This group was randomly split into training, validation, and test data sets. The images were passed through a detection model to crop around the scaphoid and were then used to train a DCNN model based on the EfficientNetB3 architecture to classify apparent and occult scaphoid fractures. Data analysis was conducted from January to October 2020.

**Exposures:**

A DCNN trained to discriminate radiographs with normal and fractured scaphoids.

**Main Outcomes and Measures:**

Area under the receiver operating characteristic curve (AUROC), sensitivity, and specificity. Fracture localization was assessed using gradient-weighted class activation mapping.

**Results:**

Of the 11 838 included radiographs (4917 [41.5%] with scaphoid fracture; 6921 [58.5%] without scaphoid fracture), 8356 (70.6%) were used for training, 1177 (9.9%) for validation, and 2305 (19.5%) for testing. In the testing test, the first DCNN achieved an overall sensitivity and specificity of 87.1% (95% CI, 84.8%-89.2%) and 92.1% (95% CI, 90.6%-93.5%), respectively, with an AUROC of 0.955 in distinguishing scaphoid fractures from scaphoids without fracture. Gradient-weighted class activation mapping closely corresponded to visible fracture sites. The second DCNN achieved an overall sensitivity of 79.0% (95% CI, 70.6%-71.6%) and specificity of 71.6% (95% CI, 69.0%-74.1%) with an AUROC of 0.810 when examining negative cases from the first model. Two-stage examination identified 20 of 22 cases (90.9%) of occult fracture.

**Conclusions and Relevance:**

In this study, DCNN models were trained to identify scaphoid fractures. This suggests that such models may be able to assist with radiographic detection of occult scaphoid fractures that are not visible to human observers and to reliably detect fractures of other small bones.

## Key Points

**Question:**

Can deep convolutional neural networks (DCNNs) detect occult scaphoid fractures not visible to human observers?

**Findings:**

In this diagnostic study of 11 838 scaphoid radiographs, the DCNN trained to distinguish scaphoid fractures from scaphoids without fracture achieved an overall sensitivity and specificity of 87.1% and 92.1%, respectively, with an area under the receiver operating curve (AUROC) of 0.955; a second DCNN, which examined negative cases from the first DCNN, achieved a sensitivity and specificity of 79.0% and 71.6% with an AUROC of 0.810. This 2-stage DCNN model correctly identified 90% of occult fractures.

**Meaning:**

These findings suggest that DCNNs can be trained to reliably detect fractures of small bones, such as scaphoids, and may be able to assist with radiographic detection of occult fractures that are not visible to human observers.

## Teaser

This diagnostic study develops and validates a deep convolutional neural network to detect both apparent and occult scaphoid fractures from radiographic images.

## Introduction

Scaphoid fractures are the most common carpal fracture,^1^ with the highest prevalence in active, young adult men.^2^ Although the estimated annual incidence is 5 scaphoid fractures per 10 000 people,^1^ the actual incidence is likely higher, as physicians fail to detect as many as 20% of scaphoid fractures on the initial radiograph.^3,4^ Failure to detect and treat scaphoid fractures can lead to detrimental consequences for patients.^2^ Occult scaphoid fractures, ie, scaphoid fractures that are difficult to detect on radiographs, are increasingly recognized as the etiology responsible for a large proportion of scaphoid nonunions.^5^ Patients with scaphoid nonunions are susceptible to further complications, including degenerative wrist arthritis, chronic wrist pain, and carpal collapse.^2^ As scaphoid fractures have the greatest prevalence in the younger working population, socioeconomic burden and productivity loss associated with scaphoid fractures are substantial; the yearly estimated economic burden for nonoperative scaphoid fracture management in the United States is approximately $58 million.^6,7^

When a scaphoid fracture is suspected, patients are managed with cast immobilization for approximately 2 weeks with interval follow-up radiograph.^8^ Although this empirical approach facilitates the management of potential occult scaphoid fractures, it leads to unnecessary immobilization for 80% of patients who never had a fracture.^9^ During these weeks, patients accumulate indirect costs, such as taking time off work and traveling long distances for clinic visits. Several cost-effectiveness analyses suggest that magnetic resonance imaging (MRI) for a suspected occult scaphoid fracture may be more cost-effective than empirical immobilization and interval radiographs.^9,10^ Nevertheless, MRI remains among the most expensive imaging modalities, and its use must be determined judiciously. An inexpensive diagnostic test that is sensitive and specific for occult scaphoid fractures may improve patient outcomes, yield cost savings from obviating the need for advanced imaging, and prevent unnecessary immobilization.

Recent advances in the field of image analysis have demonstrated that computer models can assist and even outperform humans in detecting features of radiographs.^11^ This takes place through a process called deep learning, whereby computers can learn features and data patterns not readily visible to the human eye.^11^ A class of deep learning neural networks commonly applied to image recognition is an algorithm called a deep convolutional neural network (DCNN).^12^ In the medical field, DCNNs have been applied to identify diabetic retinopathy,^13^ accurately discriminate osteoporosis from nonosteoporosis,^12^ enhance detection of pulmonary tuberculosis,^14^ classify skin cancers,^15^ and identify fractures.^16^ Given that physicians are unable to detect 1 in 5 scaphoid fractures on radiographs, a DCNN that assists physicians with identifying scaphoid fractures would improve patient outcomes.^3,4^ To our knowledge, no study has tried to construct a DCNN that can identify radiographically occult fractures in any bone. In this study, we aimed to create a DCNN that could reliably detect both apparent and occult scaphoid fractures using plain radiographs. We hypothesized the DCNN would detect occult scaphoid fractures with greater than 70% sensitivity and specificity. Given that unaided identification of occult scaphoid fractures on plain radiographs is extremely difficult or nearly impossible, a 70% detection accuracy would have clear clinical consequences.

## Methods

### Data Set

Hand radiographs from Chang Gung Memorial Hospital (CGMH) and Michigan Medicine (MM) between January 2001 and December 2019 were collected in Digital Imaging and Communications in Medicine (DICOM) format. Posteroanterior or scaphoid view hand radiographs querying for scaphoid fractures in adults older than 18 years were included. A group of senior musculoskeletal radiologists provided final image interpretations. Radiographs with ambiguous or conflicting reports were reviewed by a hand surgeon (K.C.C.), and final diagnoses were made based on the surgeon's interpretation, subsequent imaging, and follow-up clinic examination notes. Any confirmatory imaging with repeated radiographs, computed tomography (CT) scans, and MRI were also obtained and reviewed to ascertain the veracity of the initial radiology reading and confirm the ground truths of occult fractures. Subsequently, the occult fracture data set was populated by compiling the injury radiograph (first radiograph) taken at these patients’ initial hospital visits after traumatic injury. Radiographs were deidentified before transmission to the investigators for model development. This study was considered exempt from regulatory review by the University of Michigan institutional review board and CGMH. It was considered exempt from informed consent because it was secondary research, for which consent is not required. This study followed the Transparent Reporting of a Multivariable Prediction Model for Individual Prognosis or Diagnosis (TRIPOD) reporting guideline for prediction model development.

**Figure 1.**
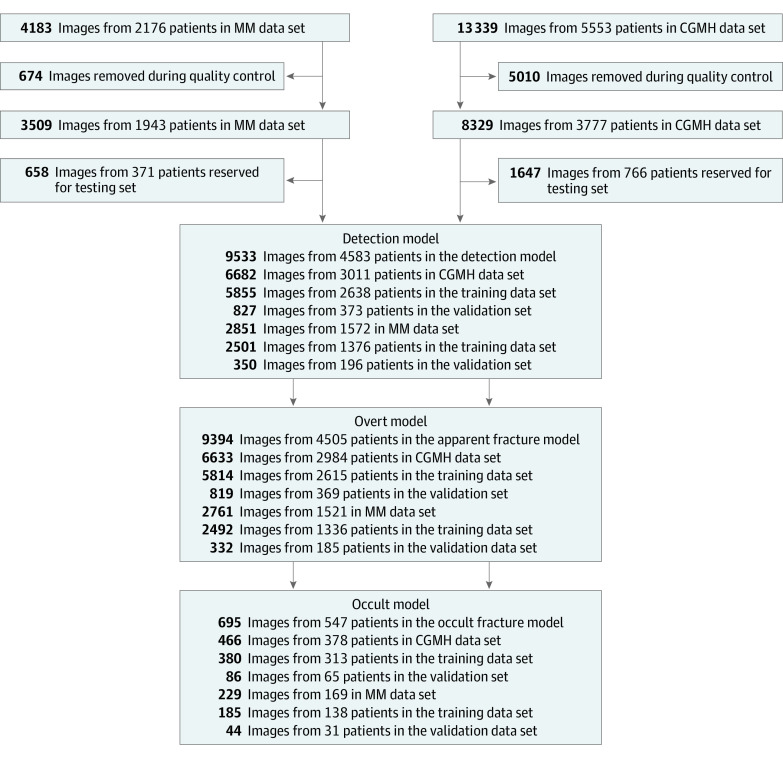
Study Workflow The data flow and training flowchart for both Chang Gung Memorial Hospital (CGMH) and Michigan Medical (MM) data sets.

In total, there were 4183 DICOM images from 2176 patients treated at MM and 13 339 DICOM images from 5553 patients treated at CGMH. The following images were excluded: (1) oblique or lateral views; (2) images with poor quality (ie, poor image detail, contrast, or inappropriate film darkness); (3) fractures older than 4 weeks; (4) images with unclear ground truth without confirmatory images or contradictory radiologic diagnoses; (5) images of chronic hand conditions with bony changes around the scaphoid; (6) images of patients with psoriatic arthritis or rheumatoid arthritis; (7) images with external immobilization (casts, splints, external fixations); and (8) images with hardware (screws, plates, wires, pins). All radiographic qualities were confirmed by 2 team members, 1 hand surgeon (A.P.Y.) and 1 machine learning engineer (Y.L.). After exclusion, 8329 images from 3777 patients treated at CGMH and 3509 images from 1943 patients treated at MM remained for inclusion. Of those, 2305 images from 1137 patients treated at both institutions were retained for the evaluation of the overall pipeline. The remaining images were used in the training and evaluation of the detection and classification models. Figure 1 depicts the image selection and data allocation process.

### Detection Model and Image Cropping

Only scaphoid images were needed for DCNN development; therefore, a scaphoid detection model based on Cascade R-CNN (Region-based Convolutional Neural Network)^17^ was trained and used to isolate the scaphoid in a bounding box in hand radiographs. A total of 2851 images from 1572 patients treated at MM and 6682 images from 3011 patients treated at CGMH were used for training. The images were standardized by image depth and were converted from grayscale to red-green-blue (RGB) color. To generalize the model, random flip, scale, random brightness, rotation (<15°), flip, resizing, and standardization were applied. Because other data augmentation techniques did not improve model performance, they were not implemented. The images were subsequently cropped around the bounding box to isolate the scaphoid.

### Classification Model

From the ImageNet Large Scale Visual Recognition Competition,^18^ several high-performing DCNNs were identified, such as AlexNet,^19^ Inception v3,^20^ and VGG-16.^21^ A recent study^22^ showed that another DCNN, EfficientNet, achieved higher accuracy than Inception v3 (95.6% vs 94.4%) with half as many parameters. For these reasons, we used the EfficientNetB3 DCNN architecture to train our model.

We developed 2 classification models. The first apparent fracture model was trained on images of radiographically apparent fractures. Because occult fractures are not readily observable by human experts and are confirmed using secondary imaging modalities, such as MRI, we developed a second DCNN that was based on the first DCNN and further adjusted using occult fracture images. This 2-stage design aimed to maximize the detection of occult scaphoid fractures.

#### Apparent Fracture Model

**Figure 2.**
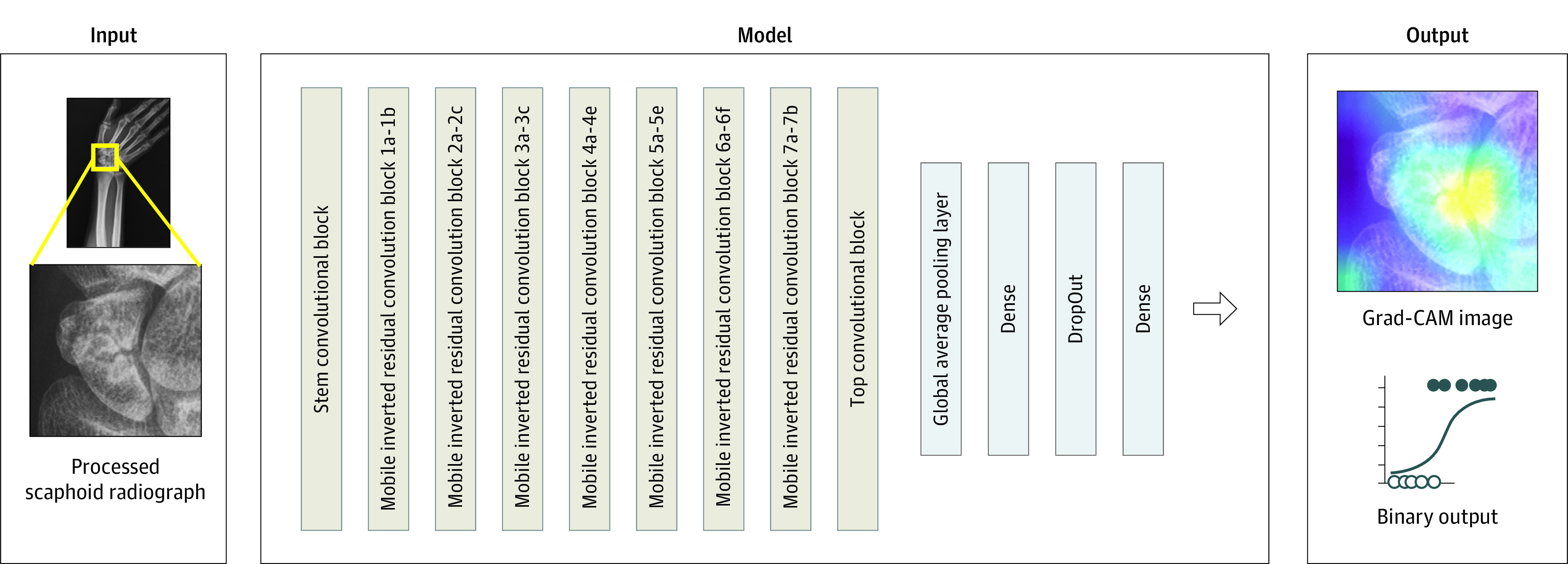
Illustration of Modeling Framework A bounding box around the scaphoid was created to isolate the scaphoid from the hand radiographs. The regions of interest were passed into the deep convolutional neural network architecture, EfficientNetB3, to train the model. Two different outputs, a binary output of presence or absence of scaphoid fracture as well as a gradient-weighted class activation mapping (Grad-CAM) image, resulted from the deep convolutional neural network.

The apparent fracture model was trained to detect radiographically evident fracture. A total of 3991 scaphoid fracture radiographs from 1911 patients (2435 images [61.0%] from CGMH and 1556 images [39.0%] from MM) and 5542 normal scaphoid radiographs from 2672 patients (4247 images [76.6%] from CGMH and 1295 images [23.4%] from MM) were used for training and validation of the apparent fracture model. Because the number of CGMH images was approximately 2.5-fold larger than the number of MM images, we oversampled MM images to balance the sampling weight. The model was trained based on MM pretrained weights using the AdamW optimizer^23^ with learning rate set as 2 × 10^−5^; weight decay, 1 × 10^−6^; and batch size, 16. The learning rate was reduced if the validation loss did not improve for 6 epochs. The training process concluded when the model performance did not improve after 15 epochs. The model outputted predicted scores for presence or absence of fracture and a gradient-weighted class activation mapping (Grad-CAM) image^24^ to visualize the possibility of a pixel to represent fracture (Figure 2). After overlaying with the original image, high probability areas were highlighted as the most likely fracture sites.

#### Occult Fracture Model

To facilitate recognition of occult fractures, the model was further trained with 49 occult fracture images from 27 patients treated at CGMH and 90 occult fracture images from 51 patients treated at MM with 417 control images from 351 patients treated at CGMH and 139 control images from 118 patients treated at MM. The ratio of case to control was 1:4. Of these, 565 images (81.3%) from 451 patients were used in training and 130 images (18.7%) from 96 patients were used for validation. Control images for the testing set were randomly picked from a larger pool of normal radiographs to better mimic real-life scenarios. The previously described image augmentation techniques were also applied to the occult model. To mitigate the small ratio of case (occult fracture) to control images, occult fracture images were oversampled once. Based on the pretrained weights of the apparent fracture model, the occult model was trained with AdamW optimizer with learning rate set as 1 × 10^−5^; weight decay, 1 × 10^−6^; and batch size, 16. The learning rate, training policy, and output types were identical to the apparent fracture model.

### Statistical Analysis

The DCNN’s fracture detection accuracy was evaluated by the area under the receiver operating characteristic curve (AUROC), sensitivity, and specificity. Optimal cutoff values for sensitivity and specificity were assessed with the Youden index, which is the point on the ROC that maximizes both sensitivity and specificity. Fracture localization accuracy was estimated as a secondary end point with Grad-CAM. Confidence intervals for sensitivity and specificity are exact Clopper-Pearson confidence intervals.^25,26^

The analysis was carried out on a DGX-1 server with ubuntu version 18.04 (Canonical) operating system using Python version 3.7 (Python Software Foundation). Four NVIDIA V100 were used to train the model. The training and inference of the detection model were completed under mmdetection version 1.0.0 and Pytorch version 1.4 framework. The bony landmarks were manually annotated using the labelme package,^27^ which was modified to accept original DICOM images. For training of the classification model, the tensorflow version 2.2 framework was used. Automatic mixed precision was used in the training process. Pydicom version 1.4.2,^28^ tensorflow, and opencv version 4.1.0 were used for image processing.

## Results

Of the 11 838 included radiographs (4917 [41.5%] with scaphoid fracture; 6921 [58.5%] without scaphoid fracture), 8356 (70.6%) were used for training, 1177 (9.9%) for validation, and 2305 (19.5%) for testing. The complete pipeline of our model was as follows. First, the detection model detected and cropped the scaphoid; then, the apparent fracture model classified apparent fractures, after which the occult fracture model identified occult fractures for images predicted as normal by the apparent fracture model. Instead of evaluating the 3 models separately, 2305 images that were not used in the training process were used to test the full pipeline. The test data set included 1379 control images (59.8%), 904 apparently fractured scaphoid images (39.2%), and 22 occult fracture images (1.0%). All test images except for 1 from the MM data set passed the scaphoid detection test. The remaining 2304 images were used for testing the pipeline (eFigure in the Supplement).

### Apparent Fracture Model Performance

**Figure 3.**
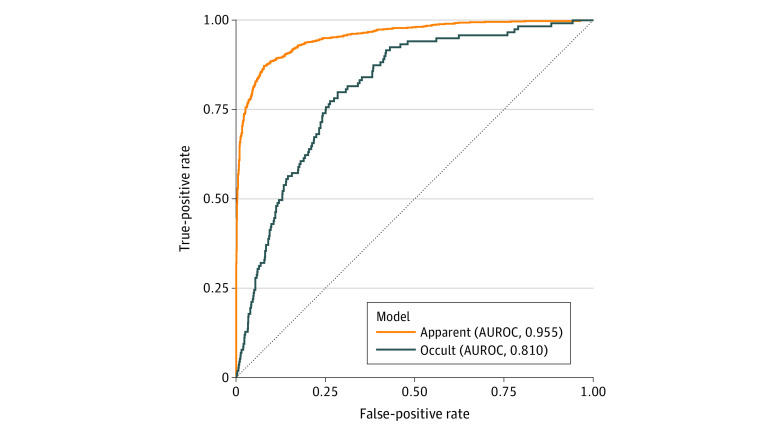
Receiver Operating Characteristic Curve of Detecting Apparent and Occult Scaphoid Fractures The model had a high area under the receiver operating characteristic curve (AUROC) for detecting both apparent and occult scaphoid fractures.

**Figure 4.**
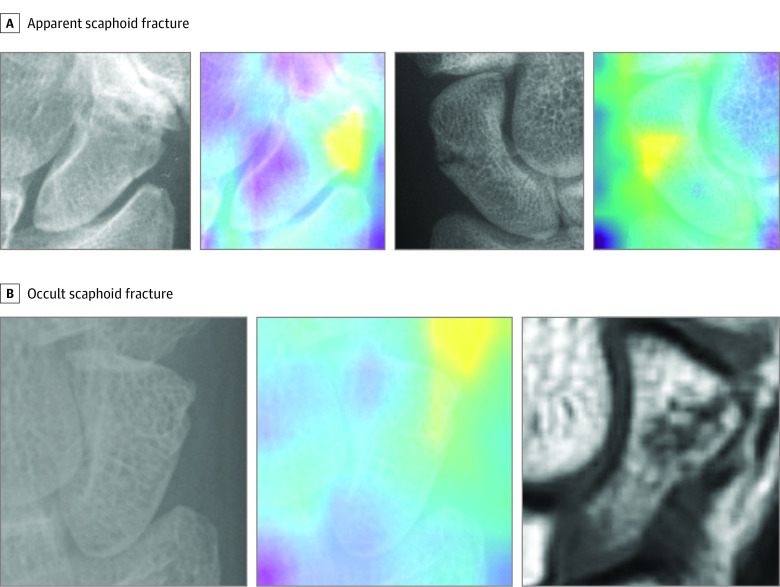
Localizing Fracture Sites Using Gradient-Weighted Class Activation Mapping (Grad-CAM) A, Example radiographs (first and third images) and Grad-CAMs (second and fourth images) for apparent scaphoid fractures. B, An example of the original radiograph (left), Grad-CAM (center), and confirmatory magnetic resonance imaging image (right) for occult scaphoid fractures. The model fracture predictions for apparent fractures (A) appear to be more precise than the predictions for occult fractures (B). For most occult fractures, the Grad-CAM images included the fracture line within the highlighted regions.

The apparent fracture model test data set included 1379 normal, 904 apparently fractured, and 22 occultly fractured scaphoid images (eTable 1 in the Supplement). The apparent fracture model correctly predicted 1271 of 1379 true-normal images (92.2%), 795 of 903 images (88.0%) of apparent fractures, and 11 of 22 images (50.0%) of occult fractures (eTable 2 in the Supplement). The model achieved an AUROC of 0.955 (Figure 3) with a sensitivity of 87.1% (95% CI, 84.8%-89.2%) and specificity of 92.1% (95% CI, 90.6%-93.5%) when tested with a set of normal and apparent scaphoid fractures. The positive predictive value (PPV) was 88.2% (95% CI, 86.1%-90.0%), and the negative predictive value (NPV) was 91.4% (95% CI, 90.0%-92.7%), with a 40.0% fracture prevalence (Table). The localization of the fracture lines was also demonstrated by Grad-CAM images (Figure 4A).

**Table.** Model Performance Detecting Apparent and Occult Scaphoid Fractures

Measure	Apparent fracture model (95% CI), %	Occult fracture model (95% CI), %^a^	Overall pipeline (95% CI), %
Sensitivity	87.1 (84.8-89.2)	79.0 (70.6-86.0)	97.2 (95.9-98.2)
Specificity	92.1 (90.6-93.5)	71.6 (69.0-74.1)	66.0 (63.4-68.5)
Positive predictive value	88.2 (86.1-90.0)	20.6 (18.7-22.8)	65.7 (64.1-67.4)
Negative predictive value	91.4 (90.0-92.7)	97.3 (96.3-98.1)	97.2 (96.0-98.1)
AUROC	0.96	0.81	NA^b^

Abbreviations: AUROC, area under the receiver operating characteristic curve; NA, not applicable.

^a^ The occult fracture model performance included both apparent and occult fractures.

^b^ Unable to calculate AUROC.

### Occult Fracture Model Performance

We tested the occult fracture model using 1390 images that were predicted as normal (119 false-negative and 1271 true-negative) by the apparent fracture model; therefore, this test data set included 1271 normal images (91.4%), 108 apparently fractured images (7.8%), and 11 occultly fractured scaphoid images (0.8%) (eTable 1 in the Supplement). Among these, the occult fracture model correctly predicted 910 of 1271 true-normal images (71.6%), 85 of 108 images (78.7%) of apparent fractures, and 9 of 11 images (81.8%) of occult fractures (eTable 2 in the Supplement). The occult fracture model achieved an AUROC of 0.810 (Figure 3) with a sensitivity of 79.0% (95% CI, 70.6%-86.0%) and specificity of 71.6% (95% CI, 69.0%-74.1%). The PPV was 20.6% (95% CI, 18.7%-22.8%), and the NPV was 97.3% (95% CI, 96.3%-98.1%) for a disease prevalence of 8.6% (Table). The model accurately localized the fracture site in Grad-CAM images when an occult fracture was detected. The predicted fracture location on the Grad-CAM images coincided with the fractures identified on follow-up CT scans and MRIs (Figure 4B).

### Overall Pipeline Performance

The overall pipeline model yielded 900 true-positive, 469 false-positive, 26 false-negative, and 910 true-negative results. It achieved a sensitivity of 97.2% (95% CI, 95.9%-98.2%) and specificity of 66.0% (95% CI, 63.4%-68.5%). The PPV was 65.7% (95% CI, 64.1%-67.4%), and the NPV was 97.2% (95% CI, 96.0%-98.1%) for a disease prevalence of 40.2% (Table). Of the 22 occult images included in the test data set, the overall model correctly identified 20 occult fracture images and misclassified 2 as normal: a 90.9% accuracy rate of detecting occult scaphoid fractures.

## Discussion

We developed a DCNN that differentiated between normal and fractured scaphoids with high specificity and sensitivity. With an AUROC of 0.955, the results of the apparent fracture model suggest that deep learning methods can detect small bone fractures, such as scaphoid fractures. Our occult fracture model also had a high accuracy, with an AUROC of 0.810, although this result must be interpreted cautiously because it originates from a test data set that included both apparent and occult fracture images. Nevertheless, given that the overall model correctly identified 20 of 22 occult fractures (90.9%), this suggests that neural networks may be able to detect fractures not visible to human observers. In addition, the model correctly localized the occult fracture sites, as seen in the Grad-CAM images (Figure 4). Although further clinical testing of the model is warranted, we propose that DCNNs may have the capacity to detect occult fractures and may outperform human observers in detecting them.

Our model pipeline consisted of 2 separate DCNNs, the apparent and occult fracture models. We intentionally implemented this 2-step process because the true prevalence of scaphoid fractures in patients presenting with acute wrist injury is reported to be between 2% and 54%,^29,30,31^ whereas occult scaphoid fracture prevalence is likely to be even lower. Because the pretest probability of occult fractures is lower, passing the image through the apparent fracture model to detect any apparent scaphoid fractures will concentrate the pretest probability for the occult fracture model, increasing its diagnostic performance. In the clinical setting, this 2-step process could not only increase the probability of detecting a true occult fracture but also increase the model’s sensitivity to rule out scaphoid fractures, especially in cohorts with low prevalence of occult scaphoid fractures, precluding the need for advanced imaging.

Neural networks have previously been applied to other musculoskeletal conditions. Several CNNs correctly classified distal radius fractures with an AUROC greater than 0.95,^32,33^ whereas others have reliably detected osteoporosis from hand radiographs.^12^ Although these illustrate the potential role of DCNNs in diagnostic radiology, a clinically meaningful application of this technology may not be possible for certain diagnoses. For example, distal radius fractures are relatively straightforward to detect on radiographs without computer vision. The standard of care for osteoporosis diagnosis is a dual-energy x-ray absorptiometry (DEXA) scan, which is comparable in cost to a hand radiograph ($125) and takes 10 minutes to administer.^34^ The reported sensitivity of a DEXA scan for osteoporosis is 98%,^35^ suggesting that a DCNN capable of detecting osteoporosis in hand radiographs with similar sensitivity may not be cost-effective compared with a DEXA scan when considering the added cost of the software. Similarly, Lindsey et al^32^ reported that the mean sensitivity and specificity of distal radius fracture detection by physicians are 80.8% and 91.5%, respectively. These estimates are likely even greater for hand surgeons and musculoskeletal radiologists, who regularly diagnose distal radius fractures. Scaphoid fractures, unlike some other musculoskeletal conditions, represent a suitable clinical dilemma for DCNNs because as many as 20% of these fractures are not readily visible to physicians on initial radiographs. This poses a unique challenge that can be overcome with computer vision.

Artificial intelligence (AI) is expected to resolve several practical challenges that radiologists confront daily. For example, approximately 40% of all inpatient imaging examinations are designated as requiring immediate attention.^36^ Such high-volume, urgent radiology interpretations can lead to observer fatigue that may diminish diagnostic accuracy, particularly in conditions, such as scaphoid fractures, for which radiographic diagnosis is already elusive. Preanalysis of radiographs with DCNNs could decrease observer fatigue and reduce missed fractures. DCNNs can make a prediction in seconds; therefore, clinical integration of these models should be effortless. Furthermore, AI can recognize complex image patterns and mathematical motifs that are not discernable to human eyes, facilitating detection of occult fractures.^16^ By contrast, trained radiologists assess new images based on knowledge of patterns learned from prior experience, which are vulnerable to human subjectivity.^37^ If DCNNs can assist physicians in reliably diagnosing occult fractures and elucidating obscure findings in other imaging modalities, immeasurable benefits to both patients and health care delivery could be achieved.

This DCNN benefited from a training data set composed of radiographs from 2 centers on 2 different continents, increasing both the diversity and power of the data set. Because the scaphoid was isolated from the hand radiographs and subsequently processed using image processing techniques, model performance was optimized while minimizing overfitting.^38^ Lastly, reliable ground truths were confirmed using conclusive radiology reports by 2 radiologists and/or follow-up imaging.

### Limitations

This study has limitations. While our long-term aim is to create a tool that enhances the standard of care for scaphoid fracture and brings tangible benefits to these patients, further refinement and validation with multiple prospective data sets are needed before this tool can be integrated into clinical workflow. Although the DCNN was trained with a sizeable data set from 2 academic institutions, incorporating radiographs from additional populations will increase the accuracy and generalizability of the DCNN. Because of their multilayered neural networks and abstractions, an inherent limitation of DCNNs is that the mechanistic steps of how the model reached its conclusions cannot be discerned.^39^ This is an important consideration because we must ensure that the model’s inferences are from the fracture site and not from irrelevant parts of the radiograph. However, the DCNN’s accuracy was demonstrated in multiple test data sets and Grad-CAM images, instilling confidence in the model’s conclusions. Indication bias is another potential limitation of our study, given that radiographs included in the training set belonged to patients with a high likelihood of scaphoid fracture. In addition, the high sensitivity and specificity of the occult model may be partially attributable to the selection of control images, which were selected based on low fracture probability predicted by the apparent fracture model. Because the apparent fracture model was highly confident that these images were normal, this may have facilitated detection of subtle features, such as occult fractures. In addition, it must be noted that the test data set for the occult fracture model included images of both apparently and occultly fractured scaphoids; therefore, the performance results for the occult fracture model are not exclusive representations of the model’s capacity to detect occult fractures. However, of the 22 occult fracture images in the test data set, 20 (90.9%) were correctly identified as having a fracture by the overall model, indicating that detection of occult fractures by DCNNs is possible. On the contrary, the relatively high false-positive rate, as evidenced by the low PPV, will likely improve with a larger training data set. But the current model’s high NPV suggests that occult scaphoid fractures are not missed, which is preferable for a clinical test, especially in low-resource settings where advanced imaging techniques are not readily available. Furthermore, lateral view radiographs were excluded to maintain a lower number of parameters, especially because most scaphoid fractures are visible on posteroanterior or scaphoid views.

## Conclusions

In this study, we developed a DCNN to identify apparent scaphoid fractures on radiographs. It achieved high sensitivity and specificity, suggesting that DCNNs can be trained to reliably detect fractures in small bones. In addition, this study found that the DCNN could detect occult fractures that are not readily visible to physicians. This enhanced diagnostic capacity can help to solve medical problems with high monetary or quality-of-life costs and improve fracture care.

## Notes

Supplement. **eTable 1.** Composition of Test Data Sets **eTable 2.** Confusion Matrices **eFigure.** Flow of Testing
