# Supplementary material for: Development and Validation of a Deep Learning Model Using Convolutional Neural Networks to Identify Scaphoid Fractures in Radiographs
Source: JAMA Netw Open. 2021 May 6;4(5):e216096. doi: 10.1001/jamanetworkopen.2021.6096 (PMC8103226; doi:10.1001/jamanetworkopen.2021.6096)
Supplement: Supplement. — eTable 1. Composition of Test Data Sets eTable 2. Confusion Matrices eFigure. Flow of Testing [file jamanetwopen-e216096-s001.pdf]

Supplemental Online Content

Yoon AP, Lee YL, Kane RL, Kuo CF, Lin C, Chung KC. Development and validation of a deep learning model using convolutional neural networks to identify scaphoid fractures in radiographs. *JAMA Netw Open*. 2021;4(5):e216096. doi:10.1001/jamanetworkopen.2021.6096

eTable 1. Composition of Test Data Sets
eTable 2. Confusion Matrices
eFigure. Flow of Testing

This supplemental material has been provided by the authors to give readers additional information about their work.

© 2021 Yoon AP et al. *JAMA Network Open.*

eTable 1. Composition of Test Data Sets

	Apparent Model	Occult Model[†]
Normal Scaphoid Images	1379	1271
Apparently Fractured Images	904	108
Occultly Fractured Images	22	11

[†] The test dataset for the occult model consisted of all images predicted as normal (not fractured) by the apparent model

© 2021 Yoon AP et al. *JAMA Network Open.*

eTable 2. Confusion Matrices

Apparent Model	Predicted Fracture	Predicted Normal
True Normal	108	1271
Apparent Fracture	795	108
Occult Fracture	11	11

Occult Model	Predicted Fracture	Predicted Normal
True Normal	361	910
Apparent Fracture	85	23
Occult Fracture	9	2

Overall Pipeline	Predicted Fracture	Predicted Normal
True Normal	469	910
Apparent Fracture	880	23 + 1[†]
Occult Fracture	20	2

[†] +1 refers to one image that the model could not detect a bounding box for, which was excluded

© 2021 Yoon AP et al. *JAMA Network Open.*

eFigure 1: Flow of Testing

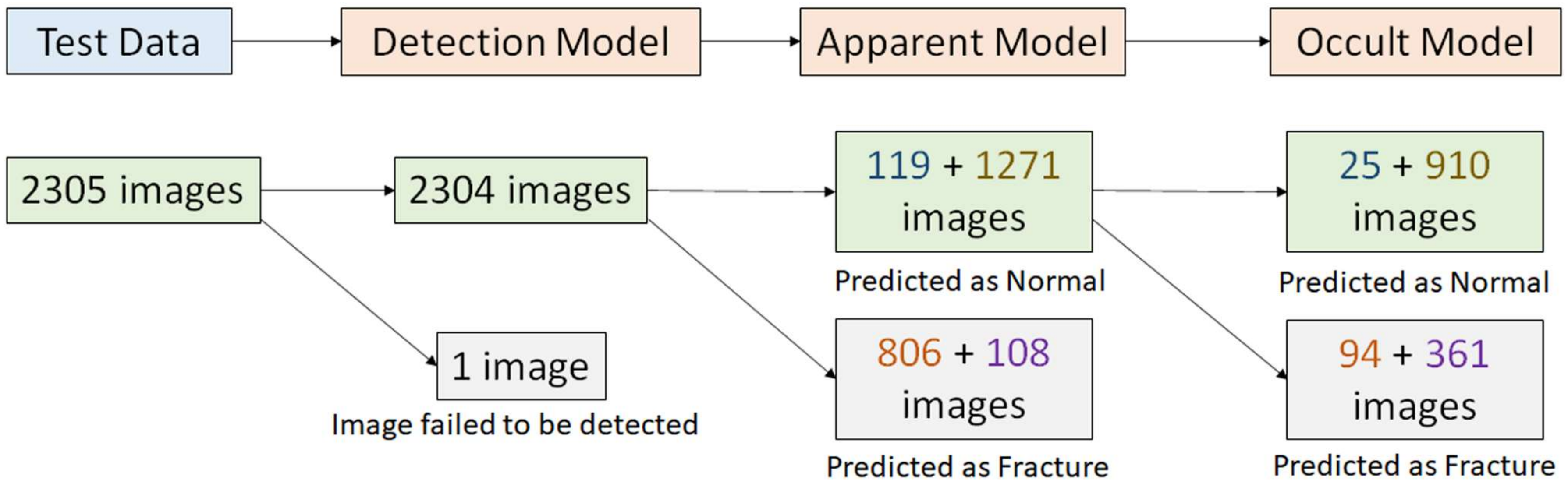

Apparent Model

N	Prediction Positive	Prediction Negative
2304	914	1390
Condition Positive	True Positive	False Negative
925	806	119
Condition Negative	False Positive	True Negative
1379	108	1271

Occult Model

N	Prediction Positive	Prediction Negative
1390	455	935
Condition Positive	True Positive	False Negative
119	94	25
ConditionNegative	False Positive	True Negative
1271	361	910

© 2021 Yoon AP et al. *JAMA Network Open.*
